# The Role of TDP-43 in Military-Relevant TBI and Chronic Neurodegeneration

**DOI:** 10.3389/fneur.2019.00680

**Published:** 2019-06-27

**Authors:** Lanier Heyburn, Venkata S. S. S. Sajja, Joseph B. Long

**Affiliations:** Blast Induced Neurotrauma Branch, Walter Reed Army Institute of Research, Silver Spring, MD, United States

**Keywords:** blast-induced brain injuries, TDP43 proteinopathy, frontotemporal lobar degeneration, TBI, Frontotemporal dementia (FTD)

## Abstract

Due largely to the use of improvised explosive devices (IEDs) and other explosives in recent military conflicts, blast-related TBI has emerged as a prominent injury sustained by warfighters. In the recent wars in Iraq and Afghanistan, traumatic brain injury (TBI) has been one of the most common types of injury sustained by soldiers and military personnel; of the ~380,000 TBIs reported in service members from 2000 to 2017, 82.3% were classified as mild (mTBI). While mTBI is associated with normal structural imaging, brief or no loss of consciousness, and rapid recovery of mental state, mTBI can nevertheless lead to persistent behavioral and cognitive effects. As in other cases of mTBI, exposure to low-level blast often does not cause immediate overt neurological effects, but may similarly lead to persistent behavioral and cognitive deficits. These effects are likely to be compounded when multiple exposures to blast and/or impact are sustained, since there is increasing evidence that multiple mTBIs can lead to chronic neurodegeneration. One common form of this deleterious outcome is frontotemporal lobar degeneration (FTLD), which is a progressive neurodegenerative process marked by atrophy of the frontal and temporal lobes, leading to frontotemporal dementia, a common form of dementia affecting behavior, cognition and language. About half of all cases of FTLD are marked by TAR-DNA binding protein (TDP-43)-positive protein inclusions. TDP-43, a DNA/RNA binding protein, controls the expression of thousands of genes and is associated with several neurodegenerative diseases including amyotrophic lateral sclerosis, Alzheimer's disease, Huntington's disease, and chronic traumatic encephalopathy. TDP-43 abnormalities have also been associated with traumatic brain injury in both pre-clinical and clinical studies. The role of TDP-43 in the manifestation of FTLD pathology in military TBI cases is currently unclear, and to date there has been only a limited number of pre-clinical studies addressing the effects of repeated blast-related mild TBI (rbTBI) in relation to FTLD and TDP-43. This review will summarize some of these findings and address the concerns and critical knowledge gaps associated with FTLD manifestation with military populations, as well as clinical findings on other forms of mTBI.

## Introduction

Traumatic brain injury (TBI) is one of the most common types of injuries sustained by military personnel. Between 2000 and 2017, TBI was reported in more than 380,000 service members, and more than 82% of these TBIs were classified as mild (mTBI) (1). mTBI is associated with brief or no loss of consciousness, normal structural imaging, and rapid recovery of memory and mental state (2), making it difficult to diagnose, and it is likely that the incidence of mTBI in military personnel is higher than reported. A specific problem for the military population is TBI caused by blast overpressure. Blast injury likely has a different primary injury mechanism than impact TBI due to different loading conditions, but there are similar downstream neurocognitive effects. However, identification of pathological processes and post-mortem studies are at a relatively nascent stage, as discussed in this review. Blast-related TBI has been a signature of Operation Iraqi Freedom and Operation Enduring Freedom, though DVBIC does not differentiate between mTBI caused by blast vs. impact, and currently this is based on retrospective analysis of a smaller cohort of warfighters (3). Concussive and sub-concussive symptoms resulting from blast exposure are often associated with unfavorable long-term clinical outcomes (4, 5). In addition to blast from improvised explosive devices (IEDs) in combat operations, repetitive mild blast overpressure exposure sustained during training and breaching exercises is also of concern. Military and law enforcement personnel who participate in training for breaching and heavy weapon systems are often exposed to multiple low-level blast exposures throughout training (6). In addition, instructors overseeing these exercises are exposed to even more low-level blasts over a longer period of time throughout their careers and report symptomatology similar to that seen following concussion (5, 6).

Though the acute symptoms of mTBI generally resolve quickly, mTBI can lead to persistent behavioral and cognitive deficits, which are thought to be compounded cumulatively following multiple mTBIs, with a “dose”-response relationship between number of injuries and symptoms (7–9). Increasingly, TBI is being causally linked to neurodegenerative diseases. The possible relationship between repetitive injury from blast overpressure exposure and chronic neurodegeneration will be discussed, with focus on clinical and preclinical findings as well as research gaps that remain to be addressed. This review is primarily focused on the emerging evidence implicating the protein marker transactive response DNA binding protein 43 kDa (TDP-43), and its role in impact and blast TBI and chronic neurodegeneration.

## Association of TDP-43 With TBI and Neurodegeneration

Among the variety of protein mediators that are of interest with respect to chronic neurodegenerative diseases, the pathological accumulation of TDP-43 has emerged as a potentially pivotal contributor. TDP-43, a DNA/RNA binding protein encoded by the *TARDBP* gene, controls the expression of thousands of different genes. In its pathological form, TDP-43 is hyperphosphorylated, ubiquitinated, cleaved into 25 and 35 kDa fragments, and mislocalized to cytoplasmic protein inclusions (10). These inclusions are found in various neurodegenerative diseases, including chronic traumatic encephalopathy (CTE), and frontotemporal lobar degeneration (FTLD) (11, 12).

Preliminary research reports have shown that repetitive mTBI may cause a type of progressive neurodegeneration now known as CTE (13, 14). Neuropathological hallmarks of CTE include brain atrophy, white matter loss, and abnormal protein accumulation (14, 15). The main protein pathology and diagnostic criterion indicative of CTE is accumulation of neurofibrillary tangles (NFTs) of hyperphosphorylated tau, a microtubule-associated protein, in neurons and glia, particularly in perivascular regions and deep in the sulci (16). In his 1928 paper on “punch-drunk” syndrome in boxers, Martland described chronic physical deficits and, importantly, cognitive dysfunction and mental deterioration (13). More recently, clinical symptoms such as irritability, aggression, depression, memory deficits, and suicidality have been described (14, 15). Because diagnosis of CTE, which is most often observed in chronic impact cases, can only be confirmed by post-mortem pathological analysis, there is a lack of consensus in the research field as to how and if the clinical features of CTE relate to the neuropathological findings, TBI and symptomology (17). There is also debate about whether CTE can result from repeated blast exposure or whether blast leads to different chronic neurodegenerative conditions (18, 19). More epidemiology studies with comorbidities of TBI and post-mortem brain pathology could inform classification of disease pathology.

In 2010, McKee et al. found that in 12 cases of CTE-diagnosed athletes, a disease previously thought of as a tauopathy, 10 individuals showed widespread TDP-43 proteinopathy (12). They later found that TDP-43 pathology progresses with the stages of CTE, which are determined based on tau NFT pathology (20). In stage I CTE, TDP-43-positive neurites can be found in the subcortical white matter and fornix; in stage II, there are isolated TDP-43-positive neurites or inclusions in the subcortical white matter, brainstem, or medial temporal lobe; in stage III, most cases of CTE show TDP-43-positive neurites in the cortex, medial temporal lobe, or brainstem; and nearly all cases of stage IV CTE have TDP-43-positive neurites and inclusions in glia and neurons in the cortex, medial temporal lobe, diencephalon, basal ganglia, brainstem, and spinal cord (16). The strong association of CTE with repetitive impact-related TBI, along with the observation of TDP-43 pathology in many cases of CTE, may point to some association between subconcussive brain trauma and TDP-43 pathology (12), though this needs to be further supported and confirmed by more independent clinical studies.

FTLD is a neurodegenerative process characterized by selective, progressive degeneration of the frontal and temporal lobes (21). There are a variety of subtypes of FTLD, characterized by the type of intracellular protein accumulations found post-mortem. The most common subtypes have accumulation of either tau (FTLD-tau) or TPD-43 (FTLD-TDP), while the remaining 10–15% have accumulation of the protein fused in sarcoma (FUS) (22).

Frontotemporal dementia (FTD), the clinical manifestation of FTLD, has been reported in military Veterans. Various epidemiological studies have found that FTD is one of the most common forms of dementia in individuals under 65 years of age, after Alzheimer's disease and vascular dementia (23–25). It is a progressive neurodegenerative disease causing changes in cognition and behavior, with three clinical variants. Behavioral variant FTD (bvFTD) is the most common form of FTD and is characterized by disinhibition, apathy, and impulsivity (26). The other forms of FTD are non-fluent/agrammatic variant primary progressive aphasia (nfvPPA), characterized by difficulty in speech production, and semantic variant primary progressive aphasia (svPPA), characterized by impaired speech comprehension (27). About 40% of FTD cases are familial, with a known family history of the disease, and are caused by a variety of genetic mutations including in the *MAPT, GRN, C9ORF72*, and *TARDBP* genes (28). The remaining 60% of FTD is sporadic, with no known family history. There is overlap between the type of protein accumulation found in the brain and the FTD variant, such that, for example, FTD-tau and FTD-TDP can both cause bvFTD (29). Though cases of FTD caused by *TARDBP* mutation are very rare, FTLD-TDP is the most common form of FTLD and is seen in both familial and sporadic FTD (28).

### Preclinical

Several preclinical studies have examined TDP-43 pathology, using various impact TBI models. These impact studies identified alterations in TDP-43 including proteolysis, phosphorylation, and formation of cytoplasmic TDP-43 granules even after only one impact. This has been shown in several studies using fluid percussion injury (FPI) or cortical contusion injury (CCI) in rodents, where TDP-43 was phosphorylated, mislocalized, and cleaved into TDP-25 and TDP-35 following injury, leading to neuronal loss (30–32). The cleavage products are also increased in astrocytes following a weight drop TBI (33). In general, these models are of moderate-to-severe impact injury rather than a mild or blast injury and involve open head surgery and a severe deformation of the brain tissue after injury. A model of repetitive mTBI, using 3 daily controlled cortical impacts, persistently increased TDP-43 expression and aggregation in the mouse cortex and hippocampus (34).

FTD-relevant behavioral and cognitive changes have also been observed in various preclinical TBI studies using single and repetitive models. Rats subjected to a single CCI display learning and memory deficits as measured by Morris water maze (30). TDP-43^A315T^ transgenic mice had significantly impaired cognition as measured by the Y maze following FPI (32). While a single mild impact causes learning impairments, repeated mTBI causes more generalized progressive impairments in cognition, learning, and behavior (35). Repetitive mild lateral FPI and weight drop models have been shown to cause learning and memory deficits, as measured by Morris water maze, novel object recognition tasks, Barnes maze, and Y maze (36, 37). Few studies of repetitive TBI and dementia have been conducted using non-rodent models, which have more similar head and brain anatomy to that of humans, leading to difficulty in scaling these studies to humans. One study using a swine model of acceleration injury, found that repeated injury causes more severe cognitive dysfunction than a single injury (38).

While the majority of studies on TBI and FTD-like symptoms have used impact injury models, there are some using blast overpressure-related TBI models. In one such study, mice exposed to low-level (2.5 psi) blast exhibited impaired memory, as measured by the novel object recognition task (39). Our research group has similarly demonstrated that blast exposure can lead to impaired learning and memory along with tau pathology (40, 41). There are few preclinical longitudinal studies investigating the relationship between blast-induced TBI, TDP-43, and FTLD. In one study, mice exposed to blast were found to develop CTE-like tau pathology and cognitive deficits (42), though this blast has high accelerative forces that are not characteristic of primary blast exposure. This study did not examine TDP-43 pathology, but it does provide a link between blast exposure and development of CTE-like symptoms. In another study, mice subjected to blast overpressure exhibited TDP-43 fragmentation and mislocalization, hallmarks of TDP-43 proteinopathy (43). A preliminary study using a rat model of blast TBI demonstrated significantly increased TDP-43 in the brain following 3 or 4 exposures to 19psi blast (44). This indicates that repetitive blast exposure may lead to disruption in TDP-43, which is likely an important aspect of neurodegenerative processes. Based upon these promising findings, further study is needed to define the relationships between brain injury sustained from blast overpressure exposure, TDP-43 proteinopathy, and the development of FTD-like symptoms. Long-term preclinical studies, particularly ones focusing on repetitive blast exposure relevant to training and breaching environment of Warfighters, are currently lacking in this field and remains an important area of research.

### Clinical

There has been limited clinical research associating chronic TBI, TDP-43 and neurodegeneration, but there is some preliminary evidence that links these factors. The clinical behavioral symptoms associated with bvFTD, such as apathy and social dysfunction, are also frequently reported in cases of TBI and CTE (45) and there have been multiple studies correlating TBI with increased risk of developing FTD. Patients with a history of TBI had earlier ages of onset for symptoms and were 3.3 times more likely to develop FTD than patients with no head trauma (30, 46, 47) In a study of Veterans diagnosed with dementia, the prevalence of TBI history was significantly greater in those with FTD than in those with other types of dementia (48). A systematic review of 47 original research studies on concussion found that multiple concussions are a risk factor for development of cognitive impairment (49). A meta-analysis of 18 journal articles on the risk of neurodegeneration after TBI found that TBI is a risk factor for development of dementia (odds ratio (OR) of 1.93), TDP-43-associated diseases (OR of 4.44), and FTD (OR of 2.97), showing an association between TBI, TDP-43, and FTD (50). While there is a growing body of literature describing observations of FTD in military Veterans (48, 51), the cause, onset, and prognosis of disease is currently unclear in military populations. Similarly, although current research trends show that brain trauma could be associated with FTD and possibly a risk factor toward sporadic FTD, a clearly defined cause-effect relationship between FTD and brain trauma is at present unclear.

A common link between repetitive impact TBI and FTD is mislocalization and aggregation of TDP-43 in neurons and glial cells. In a study of human subjects who sustained a single moderate/severe TBI, TDP-43 expression was increased in the cytoplasm, though no increase in TDP-43 phosphorylation was observed (52). A case study of a woman who sustained a single severe TBI found that she subsequently developed symptoms of dementia as well as intraneuronal TDP-43 inclusions in the frontal and temporal lobes (53). The existing reports on TDP-43 pathology following TBI are limited in both scope and number of patients, and further examination of TDP-43 in various TBI populations is necessary to establish a causal link between TBI and TDP-43 pathology.

In cases of CTE with severe TDP-43 pathology, the pattern of TDP-43 expression mimics the pattern of expression seen in FTLD-TDP, with TDP-43 proteinopathy found in all layers of the cortex, particularly in layer II, and in the dentate fascia of the hippocampus (12, 16). Additionally, among individuals with a history of repetitive mTBI who were diagnosed with CTE, 6% were diagnosed with CTE-FTLD. Of these, half had FTLD-TDP and half had FTLD-tau (20). This demonstrates that brain trauma may be a risk factor contributing to chronic neurodegeneration, potentially mediated by TDP-43 proteinopathy.

Longitudinal studies which comprehensively monitor blast overpressure exposure and resultant chronic debilitations are currently lacking in the clinical literature. In a study of individuals with a history of repetitive mTBI, 16 of 21 military Veterans were diagnosed with CTE, three of whom were exposed to blast from IEDs (20). Of those three, one was found to have TDP-43 pathology in the brain (20). Similarly, another study of military Veterans with a history of blast exposure and/or concussion found tau pathology in the brain (42). In such studies of blast-exposed Veterans, it is difficult to ensure that the subjects have only been exposed to blast and do not have a history of impact TBI. The individuals in the McKee et al. study with a CTE diagnosis and history of IED blast exposure also had a history of playing high school football and/or motor vehicle accidents (20), and therefore likely had sustained impact mTBI(s). Likewise, in the Goldstein et al., study, the military Veterans had a history of “blast exposure and/or concussive injury” (42) and therefore, it is impossible to attribute the findings to blast exposure itself. The current state of blast/neurodegeneration literature is greatly limited by a small number of subjects and many confounding factors. There have been studies showing a divergence in CTE diagnosis and blast exposure. One study reported a unique pathological finding in chronic blast-exposed Veterans: significant perivascular astrogliosis and glial scarring at boundaries between the brain and CSF and between the gray and white matter (18). This pathology was not found in individuals with chronic impact TBI, indicating that it might be a unique consequence of blast-related brain injury (18). Further, this glial scarring is in the absence of any tau pathology, which is the current diagnostic hallmark of CTE (19). This lack of consensus of whether blast exposure can lead to CTE is an important knowledge gap to be addressed.

## Summary

While there is ample evidence that multiple impact head traumas resulting in TBIs can lead to neurodegeneration, longitudinal studies addressing Warfighter needs in this domain are currently lacking, despite the emergence of evidence in Veteran populations that FTD is a primary concern. Studies targeting mechanisms underlying the pathogenesis of neurodegeneration using pre-clinical models addressing this Warfighter population are largely non-existent in the literature. Table 1 summarizes the work that has been conducted in this field to date and illustrates pertinent research gaps to be addressed in the future, including a need for more clinical research on blast-related TBI and independent studies to demonstrate CTE pathology. Although a number of studies have focused on repetitive impact TBI experienced by civilians, they generally do not include military populations and tend to not differentiate between TBI resulting from impact and blast. Moreover, those that do address blast exposure generally rely on self-reported history of blast exposure, which creates shortcomings due to recall and diagnostic error. There is also an increasingly recognized need to study multiple repetitive exposures to low-level blast such as that experienced by breachers and heavy weapons systems users. While some studies have shown associations between TDP-43, TBI, and neurodegeneration, more studies are needed to establish this paradigm. In terms of preclinical work, there has been little research on repetitive blast exposure and its effects on TDP-43 and cognitive impairment. These research gaps are areas of future study in our laboratory, using appropriate animal models. Filling these gaps will help to understand how military personnel are particularly vulnerable to neurodegenerative processes and help identify areas of possible intervention.

**Table 1:** Summary of previous research conducted on TBI, TDP-43, and FTD.

	**Clinical TBI**		**Preclinical TBI**	
**Cause/evidence of disease progression**	**Civilian TBI**	**Military TBI (blast exposure studies only)**	**Models of civilian TBI**	**Models of blast exposure**
FTD/FTD-like behavioral deficits	• History of TBI is linked to FTD (30, 47) TBI history leads to earlier-onset bv FTD (46) • Veterans with dementia: TBI prevalence higher in those with FTD than other dementias (48) • Meta-analysis: TBI is a risk factor for FTD with an odds ratio of 4.44 (50)	• Military personnel exposed to blast showed a dose-response relationship between number of exposures and symptoms (9) • **More clinical studies are needed on blast-specific injury**	• Repetitive impact TBI causes cognitive and behavioral deficits (34–38) • Repeated injury causes more severe cognitive dysfunction than single injury (38) • TDP-43^A315T^ transgenic mice have impaired cognition following TBI (30, 32)	• Mice exposed to low-level blast exhibited impaired memory (39) • **Preclinical studies on repetitive bTBI are lacking**
CTE/CTE-like symptoms	• Repetitive mild TBI was associated with CTE (12, 14, 20) • **More independent studies are lacking** • Multiple concussions are a risk factor for cognitive impairment (49)	• Veterans exposed to blast can develop CTE* (20, 42) ^*^subjects also had history of impact TBI	• Repetitive impact injury in mice causes CTE-like neuropathologic changes (34)	• **Lack of preclinical studies linking repetitive blast and CTE neuropathology**
TDP-43 proteinopathy	• Repetitive mild TBI leads to TDP-43 proteinopathy, including protein inclusions, cytoplasmic mislocalization, phosphorylation, and cleavage (12, 20, 32) • A single TBI causes increased expression and intraneuronal inclusions of TDP-43 (52, 53) • TBI is a risk factor for TDP-43 associated diseases with an odds ratio of 1.93 (50)	• CTE is found in humans exposed to blast from IEDs* (20) ^*^subjects also had history of impact TBI	• Repetitive impact TBI leads to TDP-43 aggregation (34) • A single impact injury causes TDP-43 pathology, including cleavage, phosphorylation, mislocalization, and cytoplasmic aggregation (30–33, 43, 54) • This proteinopathy is exacerbated in transgenic mice (32, 54)	• Single blast lead to TDP-43 cleavage (43) • **Lack of preclinical studies on repetitive blast and TDP-43 proteinopathy**

## Author Contributions

LH wrote the manuscript. VS and LH structured the manuscript. VS and JL edited the manuscript.

### Conflict of Interest Statement

The authors declare that the research was conducted in the absence of any commercial or financial relationships that could be construed as a potential conflict of interest.

## References

[1] Defense and Veterans Brain Injury Center DoD Numbers for Traumatic Brain Injury: Worldwide Totals (2018). Available online at: https://dvbic.dcoe.mil/dod-worldwide-numbers-tbi

[2] Department of Defense and Veterans Affairs Management of Concussion/Mild Traumatic Brain Injury. Washington, DC (2009).

[3] MacGregor AJ Dougherty AL Galarneau MR. Injury-specific correlates of combat-related traumatic brain injury in operation Iraqi freedom. J Head Trauma Rehabil. (2011) 26:312–8. 10.1097/HTR.0b013e3181e94404 20808241

[4] Mac Donald CL Barber J Jordan M Johnson AM Dikmen S Fann JR. Early clinical predictors of 5-year outcome after concussive blast traumatic brain injury. JAMA Neurol. (2017) 74:821–9. 10.1001/jamaneurol.2017.0143 28459953 PMC5732492

[5] Carr W Polejaeva E Grome A Crandall B LaValle C Eonta SE. Relation of repeated low-level blast exposure with symptomology similar to concussion. Head Trauma Rehabil. (2015) 30:47–55. 10.1097/HTR.0000000000000064 24901327

[6] Kamimori GH Reilly LA LaValle CR Olaghere Da Silva UB Occupational overpressure exposure of breachers and military personnel. Shock Waves. (2017) 27:837–47. 10.1007/s00193-017-0738-4

[7] McKee A. C Meghan E. R. Military-related traumatic brain injury and neurodegeneration. Alzheimers Dement. (2014) 10:S242–53. 10.1016/j.jalz.2014.04.003 24924675 PMC4255273

[8] Nelson NW Davenport ND Sponheim SR Anderson CR Chapter 32: blast-related mild traumatic brain injury. In: Kobeissy FH, editor. Brain Neurotrauma:Molecular, Neuropsychological, and Rehabilitation Aspects. Boca Raton, FL: CRC Press; Taylor and Francis (2015). Available online at: https://www.ncbi.nlm.nih.gov/books/NBK299235 26269865

[9] Kontos AP Kotwal RS Elbin RJ Lutz RH Forsten RD Benson PJ. Residual effects of combat-related mild traumatic brain injury. J Neurotrauma. (2013) 30:680–6. 10.1089/neu.2012.2506 23031200

[10] Neumann M Sampathu DM Kwong LK Truax AC Micsenyi MC Chou TT. Ubiquitinated TDP-43 in frontotemporal lobar degeneration and amyotrophic lateral sclerosis. Science. (2006) 314:130–3. 10.1126/science.1134108 17023659

[11] Baralle M Buratti E Baralle FE. The role of TDP-43 in the pathogenesis of ALS and FTLD. Biochem Soc Trans. (2013) 41:1536–40. 10.1042/BST20130186 24256250

[12] McKee AC Gavett BE Stern RA Nowinski CJ Cantu RC Kowall NW. TDP-43 proteinopathy and motor neuron disease in chronic traumatic encephalopathy. J Neuropathol Exp Neurol. (2010) 69:918–29. 10.1097/NEN.0b013e3181ee7d85 20720505 PMC2951281

[13] Martland HS Punch drunk. JAMA. (1928) 91:1103–7.

[14] McKee AC Cantu RC Nowinski CJ Hedley-Whyte ET Gavett BE Budson AE Chronic traumatic encephalopathy in athletes: progressive tauopathy following repetitive head injury. J Neuropathol Exp Neurol. (2009) 68:709–35. 10.1097/NEN.0b013e3181a9d503 19535999 PMC2945234

[15] Stern RA Riley DO Daneshvar DH Nowinski CJ Cantu RC McKee AC. Long-term consequences of repetitive brain trauma: chronic traumatic encephalopathy. PMandR. (2011) 3:S460–7. 10.1016/j.pmrj.2011.08.008 22035690

[16] McKee AC Stein TD Kiernan PT Alvarez VE. The neuropathology of chronic traumatic encephalopathy. Brain Pathol. (2015) 25:350–64. 10.1111/bpa.12248 25904048 PMC4526170

[17] Asken BM Sullan MJ DeKosky ST Jaffee MS Bauer RM. Research gaps and controversies in chronic traumatic encephalopathy. JAMA Neurol. (2017) 74:1255–62. 10.1001/jamaneurol.2017.2396 28975240

[18] Shively S Horkayne-Szakaly I Jones RV Kelly JP Armstrong RC Perl DP Characterization of interface astroglial scarring in the human brain after blast exposure: a post-mortem case series. Lancet Neurol. (2016) 15:944–53. 10.1016/S1474-4422(16)30057-6 27291520

[19] Perl DP Iacono D Rhodes H Lack of evidence of chronic traumatic encephalopathy in some blast-exposed post-deploted service members with prominent behavioral/neurologic symptomatology who commit suicide. In: MHSRS. Kissimmee, FL (2018).

[20] McKee AC Stern RA Nowinski CJ Stein TD Alvarez VE Daneshvar DH. The spectrum of disease in chronic traumatic encephalopathy. Brain. (2013) 136:43–64. 10.1093/brain/aws307 23208308 PMC3624697

[21] Mackenzie IR Neumann M Bigio EH Cairns NJ Alafuzoff I Kril J. Nomenclature for neuropathologic subtypes of frontotemporal lobar degeneration: consensus recommendations. Acta Neuropathol. (2009) 117:15–8. 10.1007/s00401-008-0460-5 19015862 PMC2710877

[22] Mackenzie IR Neumann M Bigio EH Cairns NJ Alafuzoff I Kril J. Nomenclature and nosology for neuropathologic subtypes of frontotemporal lobar degeneration: an update. Acta Neuropathol. (2010) 119:1–4. 10.1007/s00401-009-0612-2 19924424 PMC2799633

[23] Harvey RJ Skelton-Robinson M Rossor MN. The prevalence and causes of dementia in people under the age of 65 years. J Neurol Neurosurg Psychiatry. (2003) 74:1206–9. 10.1136/jnnp.74.9.1206 12933919 PMC1738690

[24] Snowden JS Neary D Mann DM. Frontotemporal dementia. Br J Psychiatry. (2002) 180:140–3. 10.1192/bjp.180.2.140 11823324

[25] Vieira RT Caixeta L Machado S Silva AC Nardi AE Arias-Carrión O. Epidemiology if early-onset dementia: a review of the literature. Clin Pract Epidemiol Ment Health. (2013) 9:88–95. 10.2174/1745017901309010088 23878613 PMC3715758

[26] Rascovsky K Hodges JR Knopman D Mendez MF Kramer JH Neuhaus J (2011). Sensitivity of revised diagnostic criteria for the behavioral variant of frontotemporal dementia. Brain. 134:2456–77. 10.1093/brain/awr179 21810890 PMC3170532

[27] Gorno-Tempini ML Hillis AE Weintraub S Kertesz A Mendez M Cappa SF. Classification of primary progressive aphasia and its variants. Neurology. (2011) 76:1006–14. 10.1212/WNL.0b013e31821103e6 21325651 PMC3059138

[28] Deleon J Bruce L Chapter 27—frontotemporal dementia. In: Daniel HG Henry LP Christine K, editors. Handbook of Clinical Neurology. Amsterdam: Elsevier (2018). p. 409–430. 10.1016/B978-0-444-64076-5.00027-2 29478591

[29] Olney N Spina S Miller BL. Frontotemporal dementia. Neurol Clin. (2018) 35:339–74. 10.1016/j.ncl.2017.01.008 28410663 PMC5472209

[30] Wang HK Lee YC Huang CY Liliang PC Lu K Chen HJ. Traumatic brain injury causes frontotemporal dementia and TDP-43 proteolysis. Neuroscience. (2015) 300:94–103. 10.1016/j.neuroscience.2015.05.013 25982564

[31] Wright DK Liu S van der Poel C McDonald SJ Brady RD Taylor L. Traumatic brain injury results in cellular, structural and functional changes resembling motor neuron disease. Cereb Cortex. (2016) 27:1–13. 10.1093/cercor/bhw254 27566977

[32] Tan XL Sun M Brady RD Liu S Llanos R Cheung S Transactive response DNA-binding protein 43 abnormalities after traumatic brain injury. J Neurotrauma. (2019) 36:87–99. 10.1089/neu.2017.5491 29901412

[33] Huang CY Lee YC Li PC Liliang PC Lu K Wang KW. TDP-43 proteolysis is associated with astrocyte reactivity after traumatic brain injury in rodents. J Neuroimmunol. (2017) 313:61–8. 10.1016/j.jneuroim.2017.10.011 29153610

[34] Zhang J Teng Z Song Y Hu M Chen C Inhibition of monoacylglycerol lipase prevents chronic traumatic encephalopathy-like neuropathology in a mouse model of repetitive mild close head injury. J Cereb Blood Flow Metabol. (2015) 35:443–53. 10.1038/jcbfm.2014.216 PMC4348384 25492114

[35] Mouzon BC Bachmeier C Ferro A Ojo JO Crynen G Acker CM. Chronic neuropathological and neurobehavioral changes in a repetitive mild traumatic brain injury model. Ann Neurol. (2013) 75:241–54. 10.1002/ana.24064 24243523

[36] Aungst SL Kabadi SV Thompson SM Stoica BA Faden AI. Repeated mild traumatic brain injury causes chronic neuroinflammation, changes in hippocampal synaptic plasticity, and associated cognitive deficits. J Cereb Blood Flow Metabol. (2014) 34:1223–32. 10.1038/jcbfm.2014.75 24756076 PMC4083389

[37] McAteer KM Corrigan F Thornton E Turner RJ Vink R. Short and long term behavioral and pathological changes in a novel rodent model of repetitive mild traumatic brain injury. PLoS ONE. (2016) 11:e0160220. 10.1371/journal.pone.0160220 27505027 PMC4978416

[38] Friess SH Ichord RN Ralston J Ryall K Helfaer MA Smith C. Repeated traumatic brain injury affects composite cognitive function in piglets. J Neurotrauma. (2009) 26:1111–21. 10.1089/neu.2008.0845 19275468 PMC2848948

[39] Tweedie D Rachmany L Rubovitch V Li Y Holloway HW Lehrmann E Blast traumatic brain injury induced cognitive deficits are attenuated by pre- and post-injury treatment with the glucagon-like peptide-1 receptor agonist, exendin-4. Alzheimers Dement. (2016) 12:34–48. 10.1016/j.jalz.2015.07.489 26327236 PMC4715981

[40] Arun P Abu-Taleb R Oguntayo S Tanaka M Wang Y Valiyaveettil M. Distinct patterns of expression of traumatic brain injury biomarkers after blast exposure: role of compromised cell membrane integrity. Neurosci Lett. (2013) 552:87–91. 10.1016/j.neulet.2013.07.047 23933206

[41] Sajja VS Hubbard WB Hall CS Ghoddoussi F Galloway MP VandeVord PJ. Enduring deficits in memory and neuronal pathology after blast-induced traumatic brain injury. Nat Sci Rep. (2015) 5:15075. 10.1038/srep15075 26537106 PMC4633584

[42] Goldstein LE Fisher AM Tagge CA Zhang XL Velisek L Sullivan JA. Chronic traumatic encephalopathy in blast-exposed military veterans and a blast neurotrauma mouse model. Sci Transl Med. (2013) 4:134ra60. 10.1126/scitranslmed.3003716 22593173 PMC3739428

[43] Yang Z Lin F Robertson CS Wang KK. Dual vulnerability of TDP-43 to calpain and caspase-3 proteolysis after neurotoxic conditions and traumatic brain injury. J Cereb Blood Flow Metabol. (2014) 34:1444–52. 10.1038/jcbfm.2014.105 24917042 PMC4158661

[44] Heyburn L Wilder DM Van Albert S Ahlers S Long J Sajja S Repetitive blast exposure leads to increased expression of TDP-43 and neuroinflammation in a rat model of blast TBI. In: Neurotrauma Symposium. Toronto, ON (2018).

[45] Tartaglia MC Hazrati LN Davis KD Green RE Wennberg R Mikulis D. Chronic traumatic encephalopathy and other neurodegenerative proteinopathies. Front Hum Neurosci. (2014) 8:30. 10.3389/fnhum.2014.00030 24550810 PMC3907709

[46] LoBue C Wilmoth K Cullum CM Rossetti HC Lacritz LH Hynan LS. Traumatic brain injury history is associated with earlier age of onset in frontotemporal dementia. J Neurol Neurosurg Psychiatry. (2016) 87:817–20. 10.1136/jnnp-2015-311438 26359171 PMC4835269

[47] Rosso SM Landweer EJ Houterman M Donker Kaat L van Duijn CM van Swieten JC. Medical and environmental risk factors for sporadic frontotemporal dementia: a retrospective case-control study. J Neurol Neurosurg Psychiatry. (2003) 74:1574–6. 10.1136/jnnp.74.11.1574 14617722 PMC1738250

[48] Kalkonde YV Jawaid A Qureshi SU Shirani P Wheaton M Pinto-Patarroyo GP. Medical and environmental risk factors associated with frontotemporal dementia: a case-control study in a veteran population. Alzheimer Dement. (2012) 8:204–10. 10.1016/j.jalz.2011.03.011 22465176

[49] Manley G Gardner AJ Schneider KJ Guskiewicz KM Bailes J Cantu RC A systematic review of potential long-term effects of sports-related concussion. Br J Sports Med. (2017) 51:969–77. 10.1136/bjsports-2017-097791 28455362 PMC5466926

[50] Huang CH Lin CW Lee YC Huang CY Huang RY Tai YC. Is traumatic brain injury a risk factor for neurodegeneration? A meta-analysis of population-based studies. BMC Neurol. (2018) 18:184. 10.1186/s12883-018-1187-0 30396335 PMC6217762

[51] Lai AX Kaup AR Yaffe K Byers AL. High occurrence of psychiatric disorders and suicidal behavior across dementia subtypes. Am J Geriatric Psychiatry. (2018) 26:1191–1201. 10.1016/j.jagp.2018.08.012 30392777

[52] Johnson V Stewart W Trojanowski JQ Smith DH Acute and chronically increased immunoreactivity to phosphorylation-independent but not pathological TDP-43 after a single traumatic brain injury in humans. Acta Neuropathol. (2011) 122:715–26. 10.1007/s00401-011-0909-9 22101322 PMC3979333

[53] Kenney K Iacono D Edlow BL Katz DI Diaz-Arrastia R Dams-O'Connor K. Dementia after moderate-severe traumatic brain injury: coexistence of multiple proteinopathies. J Neuropathol Exp Neurol. (2018) 77:50–63. 10.1093/jnen/nlx101 29155947 PMC5939622

[54] Wiesner D Tar L Linkus B Chandrasekar A Olde Heuvel F Dupuis L. Reversible induction of TDP-43 granules in cortical neurons after traumatic injury. Exp Neurol. (2018) 299:15–25. 10.1016/j.expneurol.2017.09.011 28941811

